# Effects of hepatocyte growth factor on porcine mammary cell growth and senescence

**DOI:** 10.37796/2211-8039.1392

**Published:** 2023-03-01

**Authors:** Chi-Ping Huang, Liang-Chih Liu, Hsin-Ling Lu, Chih-Rong Shyr

**Affiliations:** a Department of Medicine, Urology Division, China Medical University and Hospital, Taichung, Taiwan; b Department of Medicine, Department of Surgery, College of Medicine, China Medical University and Hospital, Taichung, Taiwan; c Department of Medical Laboratory Science and Biotechnology, China Medical University, Taichung, Taiwan; d eXCELL Biotherapeutics INC., Taichung, Taiwan

**Keywords:** Primary, Mammary, Xenogeneic cell, Xenoantigen

## Abstract

**Background:**

The porcine mammary glands share morphological and physiological similarities with human ones, making primary porcine mammary cells (PMC) suitable for biomedical research and a potential cellular therapeutic for breast cancer xenogeneic cell immunotherapy. Primary cells isolated from tissues remain the physiological functions of origin tissues but their self-renewal ability is restricted and cells acquire senescence during in vitro expansion. To overcome these drawbacks, here we sought to establish an approach to efficiently increase PMC’s in vitro growth. We studied the effects of the hepatocyte growth factor (HGF) to maintain the expansion capacity of porcine mammary cells and identify the possible mechanisms.

**Purpose:**

HGF could allow for the increase in vitro proliferation capacity of primary epithelial cells isolated from tissue samples. To effectively produce cells for biomedical research and xenogeneic cell therapy, we planned to study the effects of HGF and its potential mechanisms of action to stimulate cell growth for PMC expansion.

**Methods:**

After HGF treatment, the growth, cell cycle, senescence and the cell marker gene expression of PMCs were analyzed in standard 10% FBS and low serum 1% FBS containing medium.

**Results:**

HGF significantly enhanced the cell proliferation by shifting the cell cycle population from G1 phase into S phase to increase cell division, reduced the senescent cells and reprogrammed gene expression profiles.

**Conclusion:**

We demonstrated that HGF could maintain the expansion capacity of PMCs by increasing cell growth and anti-senescence capability, suggesting its potential application in optimizing the long-term culture of primary cells. Adding a specific growth factor such as HGF in culture allows enhanced expansion of heterogeneous cell populations from normal porcine mammary glandular tissues in vitro. We believe that this cell culture approach will efficiently provide cells for studying mammary cell function and supply cells for therapeutic uses.

## 1. Introduction

Primary cell cultures obtain cells freshly and directly from dissected human or animal organ tissues and maintain them for growth in vitro [1]. Primary cells most closely represent the tissue of original sources because the cells are collected directly and derived from tissue and cultured without transformation and genetic modification. Cells expanded from primary cell culture more closely replicate the physiology of biological systems in original tissues and contain relevant biological functions. Consequently, they act in a similar way to the in vivo state and exhibit normal physiology. For this reason, primary cells offer excellent biological model systems for studying the normal physiology and biochemistry of cells (e.g., biological functions, aging, signaling and metabolic studies), as well as the effects of drugs and toxic compounds on the cells [2]. In addition, primary cells, unlike immortalized cells, maintain their biological identity and can only propagate for few generations in vitro. For example, mammary cells from mammary glands are used to study mammary gland development, function and maturation as well as breast cancer carcinogenesis. In addition, animal cells from different tissues could also be used as a therapeutic agent to treat degenerative disease and cancers [3–6]. Access to human tissues for research and product development is limited [7], but lab animals and food producing animals are abundant sources for tissues. Primary cells are ubiquitous across biomedical research and have been used in cell biology, virology, cancer research, drug screening, toxicity testing, vaccine production, genetic engineering, tissue and organ replacement, cell therapy, and other applications.

The mammary gland consists of parenchyma (glandular epithelial cells surrounded by myoepithelial cells and duct epithelial cells) and stroma (extracellular matrix, adipocytes, fibroblasts, immune cells, and vascular endothelial cells). The human mammary gland composes a compound tubuloalveolar structure with luminal epithelial cells that are surrounded by myoepithelial cells to form an alveolus [8]. Alveoli join to form lobules that connect through lactiferous ducts that drain into openings in the nipple [8]. Porcine and human mammary glands share similarities in the development process from embryogenesis through puberty and anatomical structure [9]. Therefore, porcine mammary cells (PMC) isolated from porcine mammary glands serve as an appropriate in vitro model for human mammary epithelial cell biology and pathology. PMCs closely recapitulate the physiological state of cells in vivo and could generate more relevant data representing living systems for researches to understand normal and pathological tissue development and functions. PMC continuing growth depends on the stem and progenitor cell population existing in the mammary epithelium tissues, which are responsible for tissue cell turnover and maintenance of injured tissues [10]. Due to the amino acid sequence difference in homolog proteins between human and porcine mammary cells, PMCs as xenogeneic cells to human and mice, could be used as an immunotherapeutic agent to treat breast cancer [4,5]. The results in a pre-clinical murine breast tumor model study found that PMCs in monotherapy and combination with chemotherapy inhibit tumor growth [6].

Primary tissue cells like PMCs are sensitive to environmental signals by growth factors for growth and survival. Growth factors such as EGF, FGF and HGF enhance cell growth in vitro. However, it is not clearly defined whether any or all these growth factors are also important for PMC growth. To investigate the effect of these factors on the growth and expansion of PMCs, we tested the effect of these growth factors on PMC growth, cell cycle and senescence as well as the expression level of PMC progenitor and differentiation marker gene. We found that Hepatocyte Growth Factor (HGF), a pleiotropic growth factor that stimulates proliferation and migration of epithelial cells and endothelial cell through the binding to the proto-oncogenic c-Met receptor [11,12], increased cell growth, altered cell division cycle, inhibited senescence and reprogrammed the cell fates.

PMCs isolated from porcine mammary glands and expanded in vitro provide a suitable cellular model for studying human mammary gland biology, physiology and pathology, an alternative approach using animal cells to solve the scarcity of human tissues. PMCs also provide a safe animal source for xenogeneic cell therapy. Prolonged growth and further expansion of these cells could be achieved using HGF through regulating important cellular and molecular functions.

## 2. Methods

### 2.1. Porcine mammary cells (PMC) isolation and culture

The porcine mammary cells (PMC) were isolated from mammary glands, which were obtained from landrace (L), Yorkshire (Y) and Duroc (D) L-Y-D hybrid sows (age between 9 and 12 month olds, unpregnant). Briefly, porcine mammary gland biopsy tissue was disinfected using povidone-iodine solution washed, and glandular structures were harvested and cut into 1–2 mm^2^ pieces and then dissociated by digestion solution which contain 0.75 mg/ml collagenase D (Gibco, Carlsbad, CA, USA) in DMEM (Dulbecco’s modified Eagle medium) and 1 mg/ml type IV collagenase (Gibco, Carlsbad, CA, USA) in Hanks’ balanced salt solution (Gibco, Carlsbad, CA, USA), respectively. The dissociated cells and tissue fragments were cultured in DMEM/Ham’s F12 medium containing 10% FBS, 10 mM HEPES and antibiotics (100U/mL penicillin, 100 mg/ml streptomycin and 5 mg/ml amphotericin B) and incubated at 37 °C in humidified atmosphere of 5% CO2. This initial passage of the primary cell culture was referred to as passage 0 (P0). Dissociated cells and outgrown cells of tissue explants were maintained in culture until they achieved 75–90% confluence. They were then either collected and cryopreserved or passaged. To separate epithelial cells from fibroblast cells, we used differential trypsinization, which removed the fibroblasts and enriched the strongly adherent epithelial cells (based on the cell morphology).

### 2.2. PMC cell growth assay

PMCs were seeded in 96-well plates at 4 × 10^3^ cell/well with different treatments. At the end of treatments, cell proliferation of PMCs was quantified using cell counting kit-8 (CCK-8 kits; Dojindo Molecular Technologies, Kumamoto, Japan) following the manufacturer’s protocol. The absorbance was measured at 450 nm with a microplate reader (Molecular Devices, Sunnyvale CA, USA). The average absorbance of the control wells (blank) was subtracted from that of the test wells with cells. All the data were then expressed as relative absorbance using the cells at day 0 or without treatments as a reference value. To determine the doubling time for each passage, cell divisions per passage were calculated as n = [logN−logN0]/log 2 where N is the absorbance of cells at harvest and N0 the absorbance of cells at seeding.

### 2.3. Cell cycle analysis

PMCs (1 × 10^5^ cells) were seeded in 6-well plates and incubated overnight. Cells were treated with HGF for 48 h. After 48 h, cells were trypsinized and fixed in 70% ethanol at 4 °C for 15 min. Centrifuge ethanol-suspended cells and remove ethanol. After two times washing with cold PBS, the cells were stained with 300 μL of staining solution (10 μg/ml Propidium iodide; and 5 U/mL RNaseA, BD Pharmingen, Franklin Lakes, NJ, USA) at 4 °C for 30 min and analyzed by the BD FACSCanto II flow cytometry (BD Biosciences, San Jose, CA, USA). Data were then analyzed by Flowjo software.

### 2.4. Senescence-associated-β-galactosidase staining

Cells were plated at 5 × 10^4^ cells per well in 12-well and treated with HGF for 3 days. Senescence-associated- β-galactosidase (SA-β-Gal) activity was evaluated with a Senescence Detection Kit (K320-250) from Biovision (BioVision, Milpitas, CA, USA) following the manufacturer’s instruction. Senescent cells were revealed as cells that were blue-stained by Bright-field microscopy. To determine the percentage of SA-βGal-positive cells, a total of 500 cells in 3 random views on each slide were counted to calculate the proportion of senescent cells in each group. Images were acquired at a magnification of 10X.

### 2.5. Western blot assay

PMCs were seeded on 10 cm dish and incubated overnight and were then treated with HGF, for 3 days. After that, cells were lysis by cold RIPA buffer containing 1 × Protease inhibitor cocktail (ab20111, Abcam, Cambridge, MA, USA). The concentration of total protein levels was measured by a BCA protein assay kit (Pierce, Thermo Scientific, Waltham, MA, USA). 40 μg of protein was separated by 10% sodium dodecyl sulfate polyacrylamide gel electrophoresis (SDS-PAGE), transferred onto to polyvinylidene difluoride (PVDF) membrane (Millipore) with a transfer apparatus (Bio-Rad, Hercules, CA, USA). PVDF membranes were blocked in 5% non-fat dry milk dissolved in TBS buffer and then incubated with primary antibodies including mouse p16INK4a (sc-166760, Santa Cruz, Santa Cruz, CA, USA) and β-Actin (C4) antibody (Santa Cruz sc47778, 1:500) overnight at 4 °C. The immunoblots were washed three times in TBS buffer for 10 min and then detected by the second antibody solution containing goat anti-mouse IgG-HRP (Santa Cruz) for p16INK4a, or goat anti-rabbit IgG-HRP (Santa Cruz) for β-Actin for 1 h in TBS buffer. The immunoblotted proteins were visualized using an enhanced Immobilon Western Chemiluminescent HRP Substrate Reagent (Millipore, Hayward, CA, USA) and detected by the Chemidoc XRS Chemiluminescent Gel Documentation Cabinet detection system (Bio-Rad).

### 2.6. Quantitative real time-PCR

For qRT-PCR analysis, PMCs were seeded in 6-well plates at 1–2x10^5^ cell/well density and total RNAs were extracted from cells by using Trizol Reagent (MRC, Cincinnati, OH, USA). Isolated RNAs were converted into cDNA by using DNA Synthesis Kit (RR037A, TAKARA, Kusatsu, Japan) via reverse transcription. PCR assays were performed using SYBR Green Dye (QIAGEN, Dusseldorf, Germany) and a quantitative real-time PCR cycler. The PCR cycle conditions were set one cycle at 95 °C for 2 min, 40 cycles of 95 °C for 5 s, and 40 cycles at 60 °C for 30 s in order and run on a BioRad CFX96 Touch Real-Time PCR Detection System. Relative mRNA expression was calculated relative to GAPDH reference gene using comparative cycle threshold analysis by the 2 (−ΔΔCT) method [13]. Primers of porcine protein C receptor (PROCR) gene were forward 5′-GCTTACCTGAAGGAGTTCCAGG-3′ and reverse 5′-CTCAGGAGGCAGCTCGCA-3′. Primers of the porcine beta casein (CSN2) gene were forward 5′-TTGATCGCCATGAAGCTCCTC-3′ and reverse 5′-AAGGCTTTCCACAGTCTCACC-3′. Primers of porcine glyceraldehyde 3-phosphate dehydrogenase (GAPDH) gene, servedas anendogenous control were forward 5′-ATGGTGAAGGTCGGAGTGAA-3′ and reverse 5′-CGTGGGTGGAATCATACTGG-3′ [14].

### 2.7. Statistical analysis

All the data are presented as mean ± standard deviation (SD). Statistical analyses were carried out using GraphPad Prism and differences between two populations (vehicle group vs HGF group) were calculated by Student’s t-test. P values < 0.05 were considered statistically significant.

## 3. Results

### 3.1. Isolation, expansion and characterization of porcine mammary cells (PMC)

**Fig. 1:**
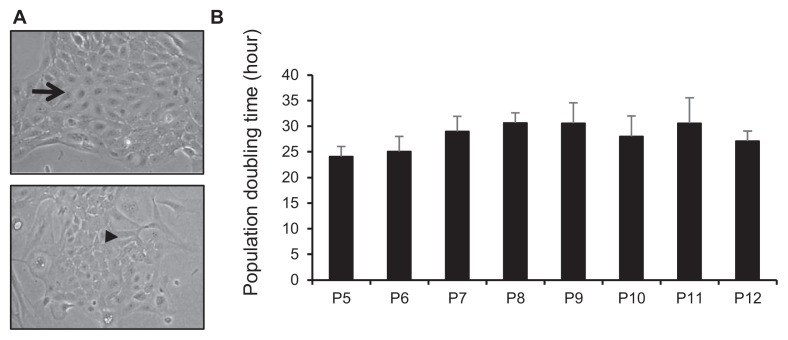
The morphology of porcine mammary cells (PMC) from adult porcine mammary glands and their cell doubling time in different passages After glandular tissue dissection, the tissues were processed by mechanical and chemical digestion into single cells and pieces of tissues. The dissociated cell and tissue explant culture reaches 90% confluent were collected and designated as passage 0 (P0) cells. The cells were then sub-cultured for further passages. (A) Representative phase contrast pictures of PMC cells. Typical cobblestone morphology of PMCs (black arrow). Lumen-like structures of PMCs (black arrowhead) (200x). (B) The cell doubling time of PMCs at different passages. Error bars represent the mean ± SD.

We successfully isolated PMCs from adult porcine mammary glands using mechanic and enzymatic processes and cultured the dissociated and explant outgrowth cells in DMEM/F12 medium for at least 12 passages. Cells displayed a monolayer of heterogeneous morphologies with most cobblestone, some oval, and luminal epithelial like shapes (Fig. 1A). Based on the cell morphology observation, most cells exhibit a tightly packed epithelial cell appearance, but some cells are spindle-shaped fibroblast like myoepithelial cells, forming islands of monolayer cells with both luminal and myoepithelial cellular appearances (Fig. 1A). PMCs were sub-cultured before they reached confluence. The expanded cells had an average doubling time of about 28 h when grown in DMEM/F12 10% FBS medium between passage 5 to 12 (Fig. 1B).

### 3.2. Hepatocyte growth factor (HGF) increases PMC cell growth

**Fig. 2:**
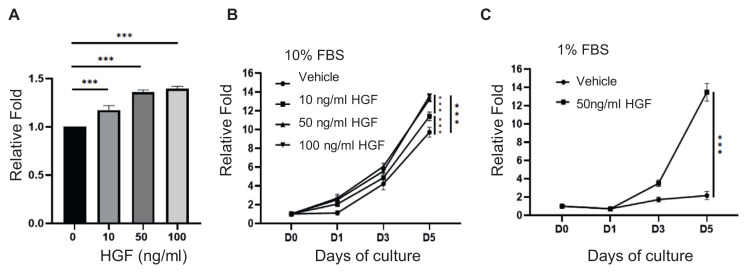
The effects of HGF on PMC growth in 10% FBS and 1% FBS medium PMCs were cultured in 96 well plates with indicated treatments for designated days before assaying for cell viability using CCK-8 assay. (A) The dose response of PMC cell growth to 0, 10, 50 or 100 ng/ml HGF in culture medium containing 10% FBS. Cell growth curves of PMCs treated with 50 ng/ml HGF in culture medium containing (B) 10% FBS or (C) 1% FBS. Error bars represent the mean ± SD. **p < 0.01. ***P < 0.005. vs vehicle control.

To identify the growth factors that could enhance the growth potential of PMCs, we selected several factors which regulate the proliferation, progenitor state, or differentiation of primary tissue cells such as HGF. We investigated the effect of HGF and its effective concentrations to promote the expansion of PMCs by culturing PMCs in medium supplemented with HGF with different concentrations for 5 days. We found that HGF promoted the growth of PMCs reaching a maximum effect at 50 ng/ml (Fig. 2A). Both the standard 10% FBS (Fig. 2B) and low serum 1% FBS (Fig. 2C) medium containing HGF could further increase cell growth, but the growth effect was more profound when cells were cultured in low serum medium with HGF compared with cells cultured in low serum medium only (Fig. 2C). These results demonstrate that 50 ng/ml HGF can effectively stimulate the growth of PMCs. Therefore, 50 ng/ml HGF was used for the following experiments.

### 3.3. Cell cycle of PMCs with or without HGF treatment in 10% and 1% FBS medium

**Fig. 3:**
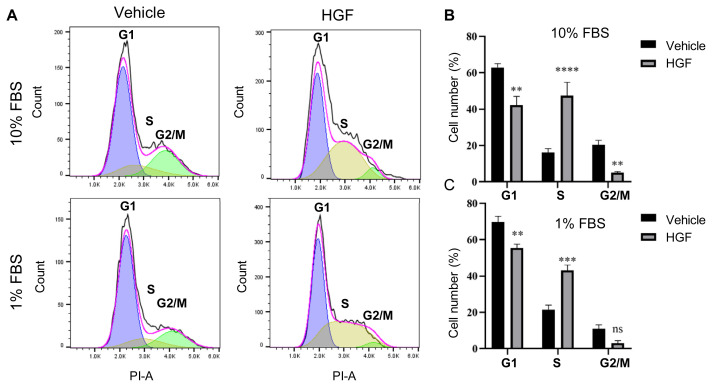
Cell cycle analysis of PMCs treated HGF in culture medium in 10% FBS and 1% FBS After 3-day treatment, the cell cycle was analyzed by propidium iodide (PI) staining and flow cytometry. (A) The representative cell cycle histogram. (B) Quantification of the percentage of cells in G1, S and G2/M phases of the cell cycle with or without HGF treatment. Error bars represent the mean ± SD derived from quantification of at least three independent experiments. ns: no significance. **p < 0.01, ***p < 0.005.

To investigate how HGF controls PMC proliferation, cell cycle events were measured. We investigated the cell cycle distribution of PMCs treated with HGF in 10% and 1% FBS medium (Fig. 3A). The cell cycle analysis of PMCs with different treatments reflected the progression of the cells through a cycle of division involving nuclear events influenced by the treatments. Sum of S and G2/M phase cells was increased by a high concentration of FBS (10% vs 1%). The cell-cycle structure of untreated PMCs is characterized as a large proportion of cells in G1 phase and reduced S and G2/M phases, especially for cells cultured in low serum medium. After, treatment, the % of S phase cells was increased by HGF at both concentrations of FBS while the fraction of cells in the G1 phase decreased, indicating the growth stimulatory activity of HGF by increasing S-phase (Fig. 3B). These results showed that HGF could enhance the proliferative abilities of PMCs during long-term expansion.

### 3.4. Senescence analysis of PMCs with or without HGF treatment in 10% and 1% FBS medium

**Fig. 4:**
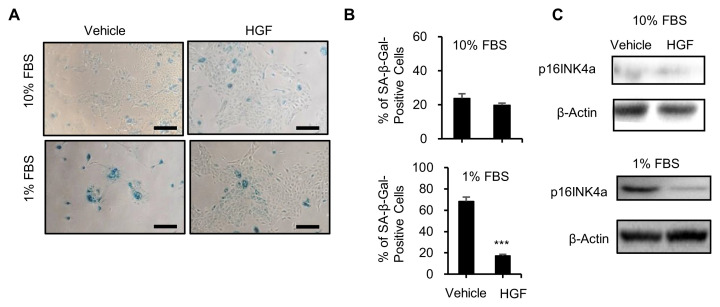
Senescence analysis of PMC treated HGF in 10% FBS and 1% FBS culture medium PMCs were treated with HGF for 3 days and then analyzed by senescence β-Gal Staining. (A) Representative bright-field images of PMCs with senescent cells showing senescence-associated β-galactosidase (SA-β-gal) activity with or without HGF treatment. Cells stained blue (SA-β-gal-positive) indicated senescence. Scale bar in (A) = 50 μm. (B) Quantification of senescent cells as (%) of β-gal-positive cells per group.***P < 0.001 vs. control treatment. (C) Immunoblot analysis of p16INK4a on PMCs in the presence or absence of HGF.

After a certain number of cell divisions, primary cells will stop dividing and enter into senesce, which is a built-in replicative limit, called the Hayflick limit [15]. The Hayflick limit describes the replicative cellular senescence that is a process affected by chromatin state, transcriptional, proteomic, and metabolomic alterations and characterized by an irreversible cessation of cell division [16,17]. After confirming that HGF promotes the growth and cell division of PMCs, we then investigated whether HGF could affect senescence of PMCs. To demonstrate the anti-senescence activity of HGF, we used senescence-associated β-galactosidase (SA-β-gal) as a maker [18]. Staining of PMCs in 10% FBS and 1% FBS with SA-β-gal indicated that PMCs were subjected to senescence in low serum medium (Fig. 4A). The results showed that SAβ-gal-positive cells were higher in low serum medium (1% FBS), indicating that the withdrawal of FBS promoted senescence of PMCs. Compared to untreated PMCs, PMCs treated with HGF showed lower SA-β-Gal-positive cells in low serum medium (1% FBS), but were not different in 10% FBS (Fig. 4B). The protein expression levels of senescence marker protein, the cyclin-dependent kinase inhibitor p16INK4a, were analyzed using western blotting. PMCs cultured in 10% FBS had lower p16INK4a expression levels than that of PMCs in low serum medium (1% FBS), in which p16INK4a decreased sharply as the cells treated with HGF, which was not observed in PMCs cultured in 10% FBS medium (Fig. 4C).

### 3.5. Expression levels of cell marker genes affected by HGF treatment measured by RTqPCR

**Fig. 5:**
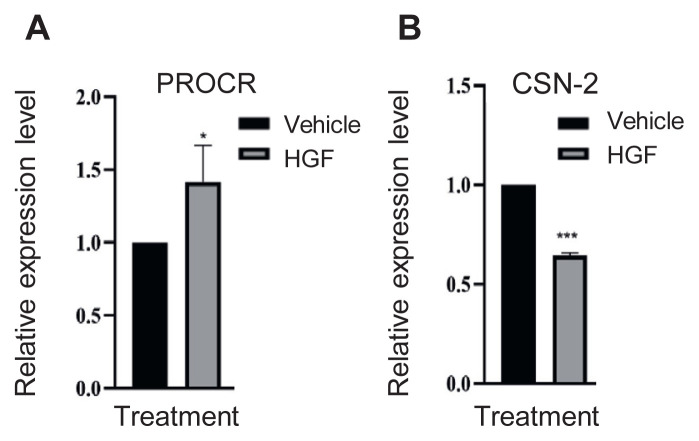
Expression level of genes involved in mammary cell stemness and differentiation in PMC cultured with or without HGF PMCs were treated with HGF for 3 d in 1% FBS medium and then cells were harvested for total RNA isolation. RT-PCR was then performed in triplicate with primers specific for porcine protein C receptor (PROCR), porcine beta casein gene (CSN2) and the housekeeping porcine GAPDH genes. (A) Relative PROCR and (B) CSN2 mRNA expression level. Data represent the mean fold-increase expression in HGF treated cells ± SD versus PMCs without treatment. * p-value <0.05; ***P < 0.005.

To further understand the effect of HGF on PMC cells, the RNA expression levels of the marker genes were measured. We used RT-qPCR to monitor progenitor or differentiation-related genes. Protein C receptor (PROCR) gene, a novel Wnt target in the mammary gland that is involved in mammary epithelium cell regenerative and multiple differentiation capacities into all lineages of the mammary epithelium [19], was highly upregulated in PMCs treated with HGF (Fig. 5A). On the other hand, the expression level of CSN2, which is responsible for the production of the β-casein protein by the lactating mammary gland, a mammary cell differentiation marker [20,21], decreased when PMCs were treated with HGF (Fig. 5B).

## 4. Discussion

We identified HGF as an active supplement in the culture of PMCs. HGF is able to increase cell growth (Fig. 2) possibly due to its capacity to increase cell cycle (Fig. 3) and suppress cell senescence (Fig. 4) as well as shift cells into stem/progenitor population (Fig. 5). Particularly, in low FBS environment, HGF could have higher activity on cell growth and anti-senescence because FBS provides a plethora of growth factors and a host of other nutrients, which could mask the effects of a single growth factor. Although the comprehensive effects of FBS could promote cell attachment, spreading, growth and proliferation, the detailed factors that induce these effects are not clear in FBS, causing variations in cell culture. However, with multiple growth factors, hormones and other biological molecules in serum, primary cells cultured in serum could become terminally differentiated or are outgrown by contaminating cells like fibroblasts. Furthermore, the demand for serum in the cell therapy industry, lot to lot variability and risks of various pathogen contaminations warrant the approaches to reduce or replace serum in cell cultures for cell expansion. Our study could provide a solution reducing or replacing the growth stimulating activities of FBS with a defined growth factor like HGF sufficient to achieve a similar cell growth.

HGF could bind to sulfated glycosaminoglycans heparan sulfate and dermatan sulfate allowing HGF to form a complex with c-Met that is able to transduce intracellular signals leading to cell division and cell migration [22]. In this study, we showed that HGF/c-met signaling was able to increase PMC cell growth with multiple actions (Fig. 2). HGF has been also shown to promote the formation of branching duct like structures by mammary gland epithelial cells in vitro, acting as a cell signaling molecule of the inducing effect of mesenchyme (or stroma) on mammary gland development [23,24]. These results indicate the potential use of HGF as a culture factor to expand PMCs.

PMCs derived directly from porcine mammary glands provide a mammary tissue specific cell model, but can only be maintained in vitro for a limited period of time with a finite short life span in conventional tissue culture conditions and thus restricted expansion capacity. Cell expansions are essential to obtain primary tissue cells because the primary cultures could be reestablished as secondary cultures that are further passaged, but eventually exhibited senescence, restricting the expansion of primary cells. The mortality stage l (MI) mechanism is activated near the end of the normal proliferative lifespan of cells and this is the process commonly viewed as in vitro senescence [25]. The limited capacity of cells to divide, after which they become senescent is known as the “Hayflick limit” [15]. Cellular senescence is characterized by a cessation of proliferation in response to serum growth factors, production of protein inhibitors of DNA synthesis and arrest in G1 [26]. Cellular senescence is the irreversible arrest of the cell cycle and inhibition of cell apoptosis due to the activation of the p53 and p16INK4a/Rb tumor suppressor pathways [27]. Furthermore, precise regulation of p16INK4a is essential to tissue homeostasis and upregulation expression of p16INK4a drives cells to enter senescence [28]. Our findings demonstrated that HGF altered the cell cycle by decreasing G1 phase and increasing S phase (Fig. 3) as well as inhibited cell senescence possibly by repressing the p16INK4a protein level (Fig. 4). Therefore, the use of growth factors like HGF could suppress the cellular senescence, which causes cells to stop cell division and lose capacity for further cell expansion and help improve the proliferation of primary cells like PMCs.

It is necessary to maintain the progenitor population of PMCs during long-term expansion to sufficiently produce PMCs in quantity for research and therapeutic purposes. We showed that HGF treatment could turn on the progenitor related PROCR gene, but inhibit differentiation marker gene CSN2 (Fig. 5) which suggests that HGF could reprogram cell state to promote the proliferation and self-renewal ability of PMCs by maintaining progenitor population. Furthermore, it is possible to show a synergistic effect of reprogramming by combining HGF with other reprogramming chemicals factors such as Y-27632 [29] as well as CHIR99021, 616452 and Forskolin [30]. Beside adding defined growth factors and chemicals to expand primary cells in two dimensional (2D) monolayer cell cultures in our study, three-dimensional (3D) cell cultures such as spheroids, organoids, scaffolds, hydrogels, organson-chips, and 3D bioprinting systems could produce cells and cellular structures, which better mimic in vivo physiology [31]. Although interventions like genetic manipulation might effectively work to expand primary cells by immortalizing them using exogenously expressed telomerase reverse transcriptase (TERT) [32] or overexpressing Yamanaka factors (Oct 3/4, Sox 2, Klf4, c-Myc) to induce pluripotency in primary cells [33], these approaches alter genetic compositions of cells, which could result in mutant cells and cell phenotype changes. Accordingly, the future direction would test the effect of HGF or other factors in 3D cell culture technologies to generate mammary cells not only as a new research tool in basic biomedicine researches and drug screening, but also as potential therapeutics to treat breast cancer [6].

## 5. Conclusion

In this study, the growth of PMC could be enhanced by HGF in 10% FBS and to a higher degree in 1% FBS medium. We identified HGF as a key factor to expand PMCs by increasing cell cycle, suppressing cell senescence in long-term culture of primary cells as well as maintaining the cells in progenitor state. Identifying the defined factors to expand primary cells is critical in order to get enough cells for both research and clinical applications. Our results indicate that HGF may support the consistent and efficient growth and expansion of porcine primary mammary cell. Due to the potential use of primary porcine mammary cells in biomedical research as well as cell-based therapy and regenerative medicine, further research is warranted to determine the factors needed to expand the cells.

## References

[1] Freshney RI Culture of Animal Cells Wiley 2005 (Chapter 12) Primary cell culture

[2] Eglen R Reisine T Primary cells and stem cells in drug discovery: emerging tools for high-throughput screening Assay Drug Dev Technol 2011 9 2 108 24 21186936 10.1089/adt.2010.0305

[3] Huang CP Yang CY Shyr CR Utilizing xenogeneic cells as a therapeutic agent for treating diseases Cell Transplant 2021 30 9636897211011995 33975464 10.1177/09636897211011995 PMC8120531

[4] Huang CP Chen CC Shyr CR Xenogeneic cell therapy provides a novel potential therapeutic option for cancers by restoring tissue function, repairing cancer wound and reviving anti-tumor immune responses Cancer Cell Int 2018 18 9 29371832 10.1186/s12935-018-0501-7 PMC5771064

[5] Huang C-P Wu C-C Shyr C-R Combination of novel intravesical xenogeneic urothelial cell immunotherapy and chemotherapy enhances anti-tumor efficacy in preclinical murine bladder tumor models Cancer Immunol Immunother 2021 70 5 1419 33 33156394 10.1007/s00262-020-02775-6 PMC8053151

[6] Huang C-P Liu L-C Chang C-C Wu C-C Shyr C-R Intratumoral xenogeneic tissue-specific cell immunotherapy inhibits tumor growth by increasing antitumor immunity in murine triple negative breast and pancreatic tumor models Cancer Lett 2022 545 115478 35902043 10.1016/j.canlet.2021.10.044

[7] Pirnay J-P Baudoux E Cornu O Delforge A Delloye C Guns J Access to human tissues for research and product development: from eu regulation to alarming legal developments in Belgium EMBO Rep 2015 16 5 557 62 25851645 10.15252/embr.201540070 PMC4428037

[8] Biswas SK Banerjee S Baker GW Kuo C-Y Chowdhury I The mammary gland: basic structure and molecular signaling during development Int J Mol Sci 2022 23 7 3883 35409243 10.3390/ijms23073883 PMC8998991

[9] Rowson-Hodel AR Manjarin R Trott JF Cardiff RD Borowsky AD Hovey RC Neoplastic transformation of porcine mammary epithelial cells in vitro and tumor formation in vivo BMC Cancer 2015 15 562 26228788 10.1186/s12885-015-1572-7 PMC4520266

[10] Visvader JE Stingl J Mammary stem cells and the differentiation hierarchy: current status and perspectives Genes Dev 2014 28 11 1143 58 24888586 10.1101/gad.242511.114 PMC4052761

[11] Bottaro DP Rubin JS Faletto DL Chan AM Kmiecik TE Vande Woude GF Identification of the hepatocyte growth factor receptor as the c-met proto-oncogene product Science (New York, NY) 1991 251 4995 802 4 10.1126/science.1846706 1846706

[12] Van Belle E Witzenbichler B Chen D Silver M Chang L Schwall R Potentiated angiogenic effect of scatter factor/hepatocyte growth factor via induction of vascular endothelial growth factor: the case for paracrine amplification of angiogenesis Circulation 1998 97 4 381 90 9468212 10.1161/01.cir.97.4.381

[13] Livak KJ Schmittgen TD Analysis of relative gene expression data using real-time quantitative pcr and the 2(-delta delta c(t)) method Methods (San Diego, CA, U S) 2001 25 4 402 8 10.1006/meth.2001.1262 11846609

[14] Zhou M Yang L Shao M Wang Y Yang W Huang L Effects of zearalenone exposure on the tgf-β1/smad3 signaling pathway and the expression of proliferation or apoptosis related genes of post-weaning gilts Toxins 2018 10 2 10.3390/toxins10020049 PMC5848150 29360780

[15] Shay JW Wright WE Hayflick, his limit, and cellular ageing Nat Rev Mol Cell Biol 2000 1 1 72 6 11413492 10.1038/35036093

[16] Chan M Yuan H Soifer I Maile TM Wang RY Ireland A Novel insights from a multiomics dissection of the hayflick limit Elife 2022 11 e70283 35119359 10.7554/eLife.70283 PMC8933007

[17] Coppé J-P Desprez P-Y Krtolica A Campisi J The senescence-associated secretory phenotype: the dark side of tumor suppression Annu Rev Pathol 2010 5 99 118 20078217 10.1146/annurev-pathol-121808-102144 PMC4166495

[18] Lee BY Han JA Im JS Morrone A Johung K Goodwin EC Senescence associated β-galactosidase is lysosomal β-galactosidase 2 5 2006 187 95 10.1111/j.1474-9726.2006.00199.x 16626397

[19] Wang D Cai C Dong X Yu QC Zhang XO Yang L Identification of multipotent mammary stem cells by protein c receptor expression Nature 2015 517 7532 81 4 25327250 10.1038/nature13851

[20] Alexander LJ Beattie CW The sequence of porcine beta-casein cdna Anim Genet 1992 23 4 369 71 1503277 10.1111/j.1365-2052.1992.tb00160.x

[21] Wartmann M Cella N Hofer P Groner B Liu X Hennighausen L Lactogenic hormone activation of stat5 and transcription of the beta-casein gene in mammary epithelial cells is independent of p42 erk2 mitogen activated protein kinase activity J Biol Chem 1996 271 50 31863 8 8943229 10.1074/jbc.271.50.31863

[22] Lyon M Deakin JA Gallagher JT The mode of action of heparan and dermatan sulfates in the regulation of hepatocyte growth factor/scatter factor J Biol Chem 2002 277 2 1040 6 11689562 10.1074/jbc.M107506200

[23] Soriano JV Pepper MS Nakamura T Orci L Montesano R Hepatocyte growth factor stimulates extensive development of branching duct-like structures by cloned mammary gland epithelial cells J Cell Sci 1995 108 Pt 2 413 30 7768990 10.1242/jcs.108.2.413

[24] Huynh HT Robitaille G Turner JD Establishment of bovine mammary epithelial cells (mac-t): an in vitro model for bovine lactation Exp Cell Res 1991 197 2 191 9 1659986 10.1016/0014-4827(91)90422-q

[25] Shay JW Wright WE Werbin H Defining the molecular mechanisms of human cell immortalization Biochim Biophys Acta 1991 1072 1 1 7 1850299 10.1016/0304-419x(91)90003-4

[26] Lee MR Lin C Lu CC Kuo SC Tsao JW Juan YN YC-1 induces G0/G1 phase arrest and mitochondria-dependent apoptosis in cisplatin-resistant human oral cancer car cells Biomedicine (Taipei) 2017 7 2 31 42 10.1051/bmdcn/2017070205 PMC5479426 28612710

[27] Kumari R Jat P Mechanisms of cellular senescence: cell cycle arrest and senescence associated secretory phenotype Front Cell Dev Biol 2021 9 645593 33855023 10.3389/fcell.2021.645593 PMC8039141

[28] LaPak KM Burd CE Themolecular balancing act of p16(ink4a) in cancer and aging Mol Cancer Res 2014 12 2 167 83 24136988 10.1158/1541-7786.MCR-13-0350 PMC3944093

[29] Zhang L Valdez JM Zhang B Wei L Chang J Xin L Rock inhibitor y-27632 suppresses dissociation-induced apoptosis of murine prostate stem/progenitor cells and increases their cloning efficiency PLoS One 2011 6 3 e18271 21464902 10.1371/journal.pone.0018271 PMC3065488

[30] Yang Z Xu X Gu C Li J Wu Q Ye C Chemicals orchestrate reprogramming with hierarchical activation of master transcription factors primed by endogenous sox17 activation Commun Biol 2020 3 1 629 33128002 10.1038/s42003-020-01346-w PMC7603307

[31] Fang Y Eglen RM Three-dimensional cell cultures in drug discovery and development SLAS Discov : Advan Life Sci R D 2017 22 5 456 72 10.1177/1087057117696795 PMC5448717 28520521

[32] Lee KM Choi KH Ouellette MM Use of exogenous htert to immortalize primary human cells Cytotechnology 2004 45 1–2 33 8 19003241 10.1007/10.1007/s10616-004-5123-3 PMC3449956

[33] Takahashi K Yamanaka S Induction of pluripotent stem cells from mouse embryonic and adult fibroblast cultures by defined factors Cell 2006 126 4 663 76 16904174 10.1016/j.cell.2006.07.024

